# Annexin A5 stabilizes matrix vesicle-biomimetic lipid membranes: unravelling a new role of annexins in calcification

**DOI:** 10.1007/s00249-023-01687-4

**Published:** 2023-11-08

**Authors:** Claudio R. Ferreira, Marcos Antônio E. Cruz, Maytê Bolean, Luiz Henrique da S. Andrilli, José Luis Millan, Ana Paula Ramos, Massimo Bottini, Pietro Ciancaglini

**Affiliations:** 1 Departamento de Química, Faculdade de Filosofia, Ciências e Letras de Ribeirão Preto da Universidade de São Paulo (FFCLRP-USP), Ribeirão Preto, São Paulo Brazil; 2 Sanford Burnham Prebys, La Jolla, CA 92037 USA; 3 Department of Experimental Medicine, University of Rome Tor Vergata, Rome, Italy

**Keywords:** Annexin A5, Phosphatidylserine, Ca^2+^, Lipid membrane, Calorimetry, Fluorescent microscopy

## Abstract

Matrix vesicles are a special class of extracellular vesicles thought to actively contribute to both physiologic and pathologic mineralization. Proteomic studies have shown that matrix vesicles possess high amounts of annexin A5, suggesting that the protein might have multiple roles at the sites of calcification. Currently, Annexin A5 is thought to promote the nucleation of apatitic minerals close to the inner leaflet of the matrix vesicles’ membrane enriched in phosphatidylserine and Ca^2+^. Herein, we aimed at unravelling a possible additional role of annexin A5 by investigating the ability of annexin A5 to adsorb on matrix-vesicle biomimetic liposomes and Langmuir monolayers made of dipalmitoylphosphatidylserine (DPPS) and dipalmitoylphosphatidylcholine (DPPC) in the absence and in the presence of Ca^2+^. Differential scanning calorimetry and dynamic light scattering measurements showed that Ca^2+^ at concentrations in the 0.5–2.0 mM range induced the aggregation of liposomes probably due to the formation of DPPS-enriched domains. However, annexin A5 avoided the aggregation of liposomes at Ca^2+^ concentrations lower than 1.0 mM. Surface pressure versus surface area isotherms showed that the adsorption of annexin A5 on the monolayers made of a mixture of DPPC and DPPS led to a reduction in the area of excess compared to the theoretical values, which confirmed that the protein favored attractive interactions among the membrane lipids. The stabilization of the lipid membranes by annexin A5 was also validated by recording the changes with time of the surface pressure. Finally, fluorescence microscopy images of lipid monolayers revealed the formation of spherical lipid-condensed domains that became unshaped and larger in the presence of annexin A5. Our data support the model that annexin A5 in matrix vesicles is recruited at the membrane sites enriched in phosphatidylserine and Ca^2+^ not only to contribute to the intraluminal mineral formation but also to stabilize the vesicles’ membrane and prevent its premature rupture.

**Supplementary Information:**

The online version contains supplementary material available at 10.1007/s00249-023-01687-4.

## Introduction

Matrix vesicles are a special class of extracellular vesicles that are thought to have a key role in both physiologic and ectopic calcification processes in virtue of their ability to accumulate calcium and phosphate ions in their lumen and initiate mineral formation (Bottini et al. 2018). Matrix vesicles were first identified by Anderson and Bonucci in the late 60’s and have been shown to bind to collagen fibrils and propagate mineralization (Bonucci 1967; Anderson 1967; Plaut et al. 2019). Lipidomic analyses have shown that the matrix vesicles’ membrane is enriched in cholesterol, phosphatidylserine and sphingomyelin in comparison to their progenitor osteogenic cells, suggesting that these vesicles are released from specialized areas of the cell membrane with a high affinity for glycosylphosphatidylinositol-anchored proteins (Simão et al. 2019; Bolean et al. 2020). In fact, the amount of tissue-nonspecific alkaline phosphatase (TNAP), a key enzyme in biomineralization, is approximately tenfold higher in matrix vesicles than in their progenitor cells, making these vesicles key regulators of calcification by controlling the extracellular ratio between inorganic phosphate and pyrophosphate (Anderson et al. 2005; Ciancaglini et al. 2010; Andrilli et al. 2022).

Proteomic studies have suggested that matrix vesicles might have a biochemical machinery dedicated to the regulation of Ca^2+^ dynamics in mineralization (Bottini et al. 2018). Several members of the annexin family have been identified in matrix vesicles, with annexin A5 being the most abundant (Balcerzak et al. 2008; Thouverey et al. 2011). Annexin A5 has a high affinity for negatively charged phospholipids, such as phosphatidylserine, and binds to these lipids in a Ca^2+^-dependent manner (Patel et al. 2005). Annexin A5 was the first annexin member to have its structure determined (Huber et al. 1990; Lin et al. 2020). The interaction of annexin A5 with negatively charged membranes has been described as a self-assembly process that produces bidimensional arrays in the presence of Ca^2+^: the protein assembles into symmetric trimers and crystallizes with an either p3 or p6 symmetry depending on both the amount of negatively charged phospholipids present in the membrane and the Ca^2+^ concentration (Gerke et al. 2005; Bouter et al. 2015). Annexin A5 has been also described to be involved in cell membrane repair via bidimensional crystallization around the defect sites (Bouter et al. 2011; Lin et al. 2020). Finally, annexin A5 has a role in apoptosis by changing the curvature of lipid membranes (Kirsch et al. 1997; Wuthier and Lipscomb 2011). These processes are of crucial importance for the function of annexin A5 as well as for its interaction with lipid membranes (Boye et al. 2018; Lin et al. 2020; Mularski et al. 2021).

The abundance of annexin A5 in the matrix vesicles’ membrane and its association with the mineral phase suggest that the protein might take part in the nucleation of calcium phosphate (Bottini et al. 2018; Plaut et al. 2019). The affinity of annexin A5 for type II and type X collagen suggests that the protein could guide the matrix vesicles to specific sites of the extracellular matrix (von der Mark and Mollenhauer 1997; Kim and Kirsch 2008; Bolean et al. 2020). A recent study suggested that Annexin A5 might also take part in Ca^2+^ uptake by matrix vesicles. Pasquarelli and co-workers recently described that the self-assembly of annexin A5 in the vesicles’ membrane may create a hydrophilic pore that can be exploited by the vesicles to accumulate Ca^2+^ in the lumen (Pasquarelli et al. 2022). However, the lack of transmembrane domains, the lower efficiency of Ca^2+^ transport compared with specific ionophores, and the fact that annexin A5 is present in higher amounts than other identified transporters in the membrane of matrix vesicles, makes it difficult to address Ca^2+^ transport as being its primary role in mineralization (Wuthier and Lipscomb 2011).

Herein, we evaluate the ability of annexin A5 to stabilize the matrix vesicles’ membrane by using liposomes and Langmuir monolayers as biomimetic models of matrix vesicles. The role of annexin A5 in the organization of lipid membranes was assessed by investigating the efficacy of the protein to bind Ca^2+^, preventing high local Ca^2+^ concentrations that would cause the disruption of negatively charged membranes (Marr et al. 2012). Since the interaction of annexin A5 with lipid membranes is majorly determined by the charge of the lipids’ polar head (Fezoua-Boubegtiten et al. 2010), although membrane fluidity through addition of cholesterol also plays a role (Jeon et al. 2010), this study was carried out by using biomimetic models made of dipalmitoylphosphatidylserine (DPPS) and dipalmitoylphosphatidylcholine (DPPC), which are simple enough to evaluate the crucial role of PS charge on such interactions. Our study supports the model that annexin A5 in matrix vesicles is recruited at the membrane sites enriched in phosphatidylserine and Ca^2+^ not only to contribute to the intraluminal mineral formation but also to enhance membrane integrity, thus avoiding premature rupture.

## Experimental procedure

### Materials

1,2-dipalmitoyl-*sn*-glycero-3-phosphatidylcholine (DPPC; purity ≥ 99%), 1,2-dipalmitoyl-*sn*-glycero-2-phosphatidylserine (DPPS; purity ≥ 99%), HEPES sodium salt (purity > 99.5%) and chloroform (purity ≥ 99%) were purchased from Sigma-Aldrich. Sodium chloride (purity > 99.5%) and calcium chloride (purity > 99%) were purchased from Merck. Methanol (purity 99.9%) was purchased from J. T. Baker. 2-(6-(7-Nitrobenz-2-oxa-1,3-diazol-4-yl)amino)hexanoyl-1-hexadecanoyl-sn-glycero-3-phosphocholine (NBD-HPC) was purchased from Avanti Polar Lipids. Ultrapure deionized water (18.2 MΩ.cm at 25ºC) produced by a Mili-Q system was used in all experiments.

### Annexin A5 expression

Annexin A5 (35 kDa) was expressed as previously described by using the pProEx.Htb.annexinV plasmid, kindly provided by Seamus J. Martin (Dublin, Ireland) (Logue et al. 2009; Bolean et al. 2015). *Escherichia coli* was used as expression system and a N-terminal poly-His tag tail sequence in the plasmid allowed for easy purification through affinity chromatography. Protein quantification was done according to Hartree in presence of SDS 2 wt% (Hartree 1972).

### Liposome preparation

Liposomes composed by pure DPPC, and mixed DPPC and DPPS in 9:1 and 4:1 molar ratios were prepared by the lipid film hydration method followed by mechanical extrusion (Bolean et al. 2015). Briefly, the lipids were solubilized in chloroform:methanol (3:1 molar ratio) solution and dried under nitrogen flow while stirring for lipid film formation. The lipid films were kept under vacuum overnight for evaporation of any residual solvent and then hydrated in HBS buffer (10 mM HEPES with 150 mM NaCl at pH 7.4) to reach a final lipid concentration of 1.5 mg/mL, for 1 h at 65 °C with periodic stirring at 10-min intervals. The resulting suspension was extruded through a 100 nm polycarbonate membrane (Millipore) by using a Liposofast extrusion system (Liposofast, Sigma-Aldrich) to obtain monodisperse large unilamellar vesicles.

### Dynamic light scattering

The hydrodynamic diameter of the liposomes was assessed by dynamic light scattering measurements. Liposomes composed by pure DPPC and mixed DPPC and DPPS at a total lipid concentration of 20 µM were dispersed in HBS buffer with increasing concentrations of Ca^2+^ (0–2 mM) in presence and absence of annexin A5 (100:1 molar ratio of lipid:protein). DLS measurements were performed in triplicate using a Nano Zetasizer (Malvern Instruments), laser wavelength of 532 nm with an angle of incidence of 173º.

### Differential scanning calorimetry

Differential scanning calorimetry measurements of liposomes were performed in a N-DSC II: Differential Scanning Calorimeter (Calorimetry Sciences Corporation). The reference used was HBS buffer pH 7.4 deaerated for 20 min, supplemented with 0.5 mM CaCl_2_ when convenient. The liposome concentration was 0.8 mM, and annexin A5 was added at a molar ratio lipid:protein of 100:1. The scanning parameters were: heating and cooling rates of 0.5 ºC/min, ranging from 10 to 70 ºC under constant pressure of 3 atm. Deconvolution of DSC data was done on OriginPro by using a Gaussian model for fitting. The R-square and adjusted R-square values considered for an acceptable fitting were each of at least 0.996.

### Langmuir monolayers preparation and surface pressure (π) *vs* area (A) measurements

Lipid monolayers consisting of either pure DPPC, mixed DPPC and DPPS at 9:1 and 4:1 molar ratios, or pure DPPS were prepared with the aid of a Langmuir trough (Insight Brazil, 216 cm^2^). First, the lipids were dissolved in chloroform:methanol (3:1 molar ratio) solution and then spread at the air/liquid interface of a subphase containing either ultrapure water from a Milli-Q system (surface tension = 72.3 mN/m and conductivity of 1.1 µS/cm, at 25 °C) or HBS buffer (10 mM HEPES pH 7.4, 150 mM NaCl, supplemented with 5 mM CaCl_2_ when necessary). The compression of the monolayers started 5 min after lipid spreading to ensure complete evaporation of solvent, with a constant rate of 0.42 mm^2^/s at 25 ± 1 °C. The changes in π were measured by a Wilhelmy plate and recorded as a function of the area occupied per molecule (A) until the collapse of the monolayer.

### Surface pressure changes as a function of time (monolayer stability)

Monolayer stability assays were carried out on preformed lipid monolayers composed by either pure DPPC, mixed DPPC and DPPS or pure DPPS at initial π = 30 mN/m. The assays were carried on subphase containing HBS buffer (10 mM HEPES pH 7.4, 150 mM NaCl, supplemented with 5 mM CaCl_2_ as needed) and annexin A5, keeping the protein to lipid molar ratio at 1:100. Annexin A5 was added to the subphases of the trough after the monolayers had reached the desired π. Π variations were acquired by a Wilhelmy plate and recorded as a function of time. The temperature was kept constant at 25 ± 1 °C.

### Fluorescence microscopy

Lipid monolayers were produced at π = 10 mN/m containing 1 mol% of NBD-HPC as fluorescent probe, both in presence and absence of CaCl_2_ (5 mM) and AnxA5 (1:100, protein:lipid molar ratio). The monolayers were imaged by using a fluorescence microscope (Olympus BX50) after complete evaporation of the solvent. The temperature was kept constant at 25 ± 1 °C.

## Results and discussion

### Role of Ca^2+^ in the stability of liposomes

**Fig. 1:**
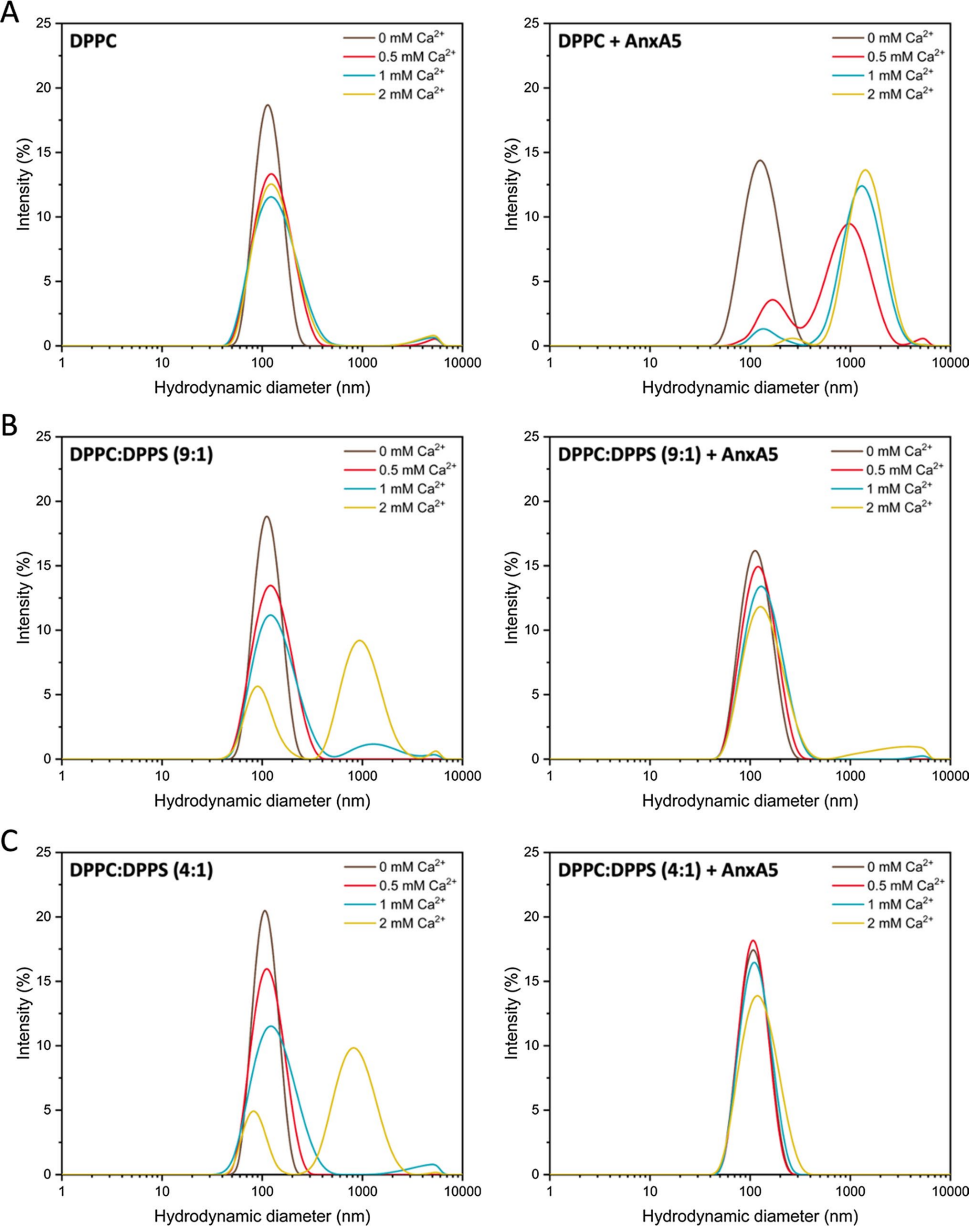
The addition of annexin A5 to DPPS-containing liposomes prevents aggregation. Distribution of the hydrodynamic diameter of liposomes made of pure DPPC (**A**) and DPPC:DPPS at a molar ratio of 9:1 (**B**) and 4:1 (**C**) dispersed in HPS buffer in the absence and in the presence of Ca^2+^ and/or annexin A5

The minimum concentration of Ca^2+^ required to trigger the aggregation of liposomes made of DPPC and DPPC:DPPS was evaluated by adding Ca^2+^ at concentrations up to 2 mM to the liposome mixtures and recording their dynamic light scattering distribution. The results showed that, in the absence of Ca^2+^, the liposomes were monodispersed with a diameter of approximately 100 nm regardless the composition. The addition of Ca^2+^ led to a slight aggregation of DPPC liposomes as suggested by the small peaks close to 5000 nm (Fig. 1A, left panel). When DPPS was added to the lipid mixture, a significant aggregation was observed at a Ca^2+^ concentration of 2 mM as shown by the strong peak at approximately 1 μm (Fig. 1B, C, left panel).

These findings agree with previously reported data, which suggested that Ca^2+^ ions trigger the destabilization of DPPS-containing liposomes. This phenomenon has been shown to be driven not only by liposomes aggregation (reversible) but also vesicle fusion (irreversible) since adding EDTA did not make liposomes to reach back their initial sizes (Marr et al. 2012). The addition of annexin A5 to the liposomes prevented Ca^2+^-induced destabilization of the vesicles containing DPPS (Fig. 1B, C, right panels), however the protein did not stabilize DPPC liposomes, and in fact enhanced destabilization (Fig. 1A, right panel).

These results suggest that annexin A5 stabilizes DPPS-containing liposomes due to specific interactions with Ca^2+^ and DPPS-containing membranes (López Cascales et al. 2006; Cruz et al. 2020). Since the destabilization of DPPS-containing vesicles by Ca^2+^ has been assigned to an increase of the lateral tension, the stabilization by annexin A5 can be explained due to the lowering of the lateral tension caused by changes on calcium binding to the membrane. Zeta potential measurements (see Supplementary Information) showed that the surface charge of the liposomes had a value of approximately – 40 mV and – 10 mV in the absence and in the presence, respectively, of Ca^2+^. Given the preferential interaction of annexin A5 with negatively charged vesicles, the observed stabilization can be related to the ordered assembly of the protein on the membrane, which did not take place in liposomes made of pure DPPC.

**Fig. 2:**
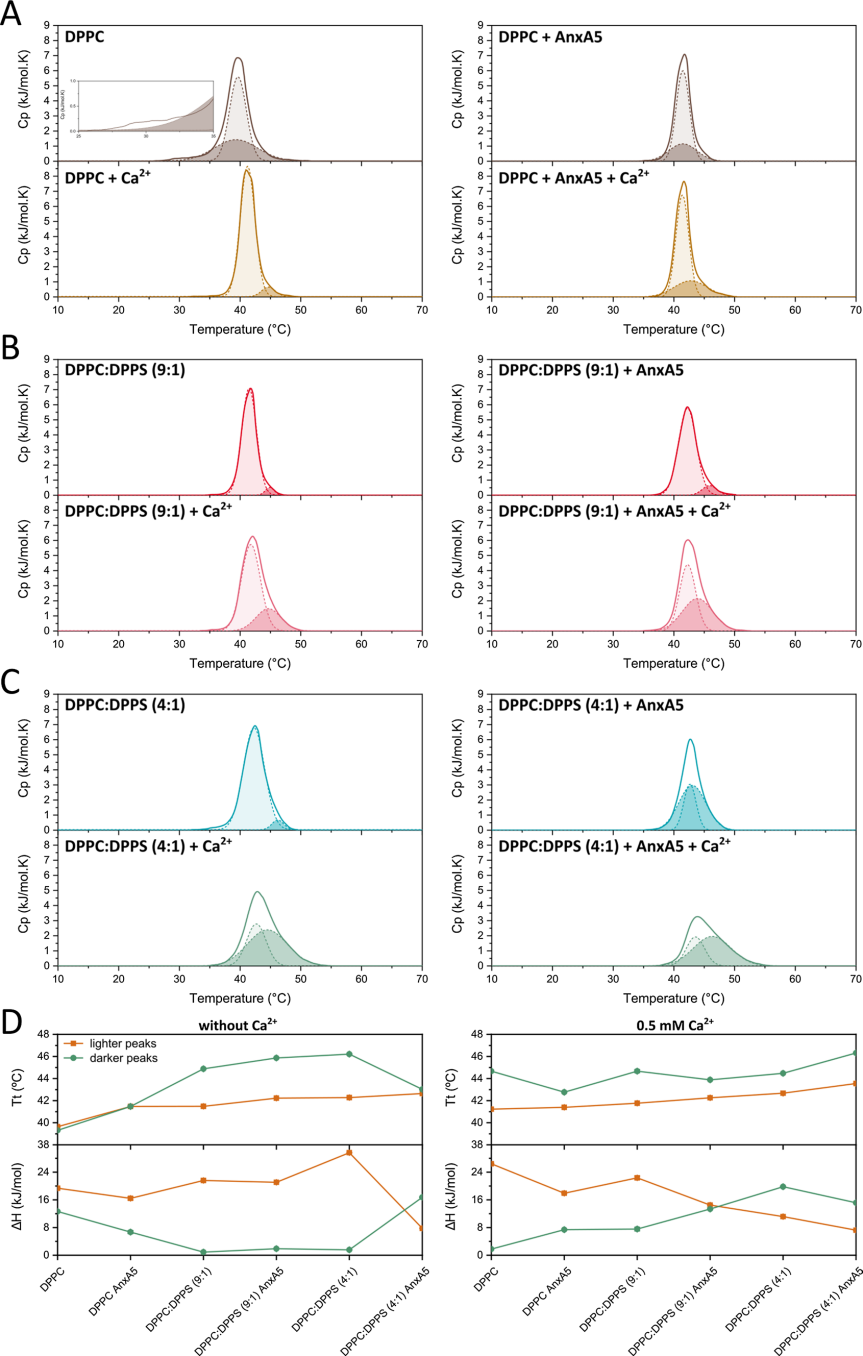
Effect of DPPS and annexin A5 on the formation of lipid domains in liposomes. Thermograms (solid lines) of lipid phase transition for liposomes made of pure DPPC (**A**) and DPPC:DPPS at a molar ratio of 9:1 (**B**) and 4:1 (**C**) in the absence and in the presence of annexin A5 and/or 0.5 mM of Ca^2+^. Deconvolution (dashed lines) has been performed to better evaluate the presence of lipid domains with higher phase transition temperature. The thermodynamic parameter extracted from the thermograms: transition temperature (Tt) and enthalpy change (ΔH) are shown in (**D**). Insert in (**A**) shows the pre-transition peak for DPPC-liposomes

To understand the nature of the stabilization/destabilization of the liposomes by Ca^2+^ and annexin A5, we carried out differential scanning calorimetry of the liposomes in the presence of the lowest Ca^2+^ concentration tested in this study (0.5 mM). The thermograms depicted the changes in the energy absorbed as heat by the liposomes during lipid phase transition, in the presence and in the absence of Ca^2+^ and annexin A5 (Fig. 2). DPPC-liposomes exhibited a small pre-transition peak at lower values of temperature (see insert in the Fig. 2A, left panel) compared to the main transition peak, in accordance with the literature (Riske et al. 2009). The pre-transition peak was slightly shifted toward lower values of temperature when compared to previously reported data, probably due to osmotic stresses at the ripple phase stage (Perkins et al. 1997; Chen et al. 2018). The pre-transition peak was absent in the presence of annexin A5, Ca^2+^, or both, indicating that annexin A5 and Ca^2+^ prevented the ripple phase organization of the lipids (Fig. 2A).

The addition of Ca^2+^ shifted the main transition peak of the liposomes to higher values of temperature both in the presence and in absence of annexin A5. Similarly, the addition of DPPS to DPPC-liposomes prevented the appearance of the pre-transition peak and shifted the main transition peak to higher values of temperature (Fig. 2B). This can be explained by the higher phase transition temperature of DPPS compared to DPPC (Gaestel et al. 1983; Wodlej et al. 2019).

The addition of Ca^2+^ broadened the phase transition band and increased the area of the secondary phase transition of the liposomes made of a mixture of DPPC and DPPS (Fig. 2B, C). This trend seemed to correlate with the amount of DPPS, suggesting that the lipid molecules undergoing a phase transition in the presence of Ca^2+^ were mostly DPPS (Fig. 2C). This effect played by Ca^2+^ over phosphatidylserine-containing membranes has been already observed for membranes made of 60% phosphatidylserine (Papahadjopoulos et al. 1974). In this study, a separate peak was observed close to 55 °C at high Ca^2+^ concentrations, in agreement with our data. The value of ΔH increased with the addition of DPPS in the liposomes only in the presence of Ca^2+^, reinforcing the putative chemically distinct nature and different organization of the domains due to the presence of the cation (Fig. 2D). The binding of Ca^2+^ to the lipid microdomains might stabilize them against the charge repulsion of the polar heads, thus enabling the formation of the microdomains. However, the binding of Ca^2+^ to the lipid polar heads has been proposed to be responsible for vesicle fusion (Marr et al. 2012). By this proposed mechanism, the binding of Ca^2+^ to the membrane affected the effective size of DPPS molecules in the bilayer but not that of DPPC molecules, so that DPPS molecules got closer to each other until the point that the lateral tension due to the repulsion of the polar heads overcomes the attraction promoted by Ca^2+^, leading to membrane rupture. This mechanism can also explain the presence of the second peak in the dynamic light scattering measurements corresponding to the formation of bigger particles in the presence of Ca^2+^ (Fig. 1B, C). Although fusion is expected to not occur at the concentrations of Ca^2+^ used for the differential scanning calorimetry analyses, the enrichment of the membranes in DPPS along with increasing Ca^2+^ concentration seemed to be the driving forces for vesicle fusion, even though it is not clear at which point the stabilizing effect of Ca^2+^ turns into destabilization. The addition of annexin A5 to DPPS-containing liposomes had an effect like that of Ca^2+^ (*i.e.*, the main transition peak was shifted to higher temperatures) (Fig. 2B, C, right panels). In the presence of Ca^2+^, the addition of annexin A5 led to an additional increase in the peaks corresponding to DPPS-rich domains, suggesting the formation of segregated domains due to the affinity of annexin A5 for negatively charged membranes (Lizarbe et al. 2013; Bolean et al. 2015, 2017).

### Role of annexin A5 and Ca^2+^ in the organization of lipids in Langmuir monolayers

**Fig. 3:**
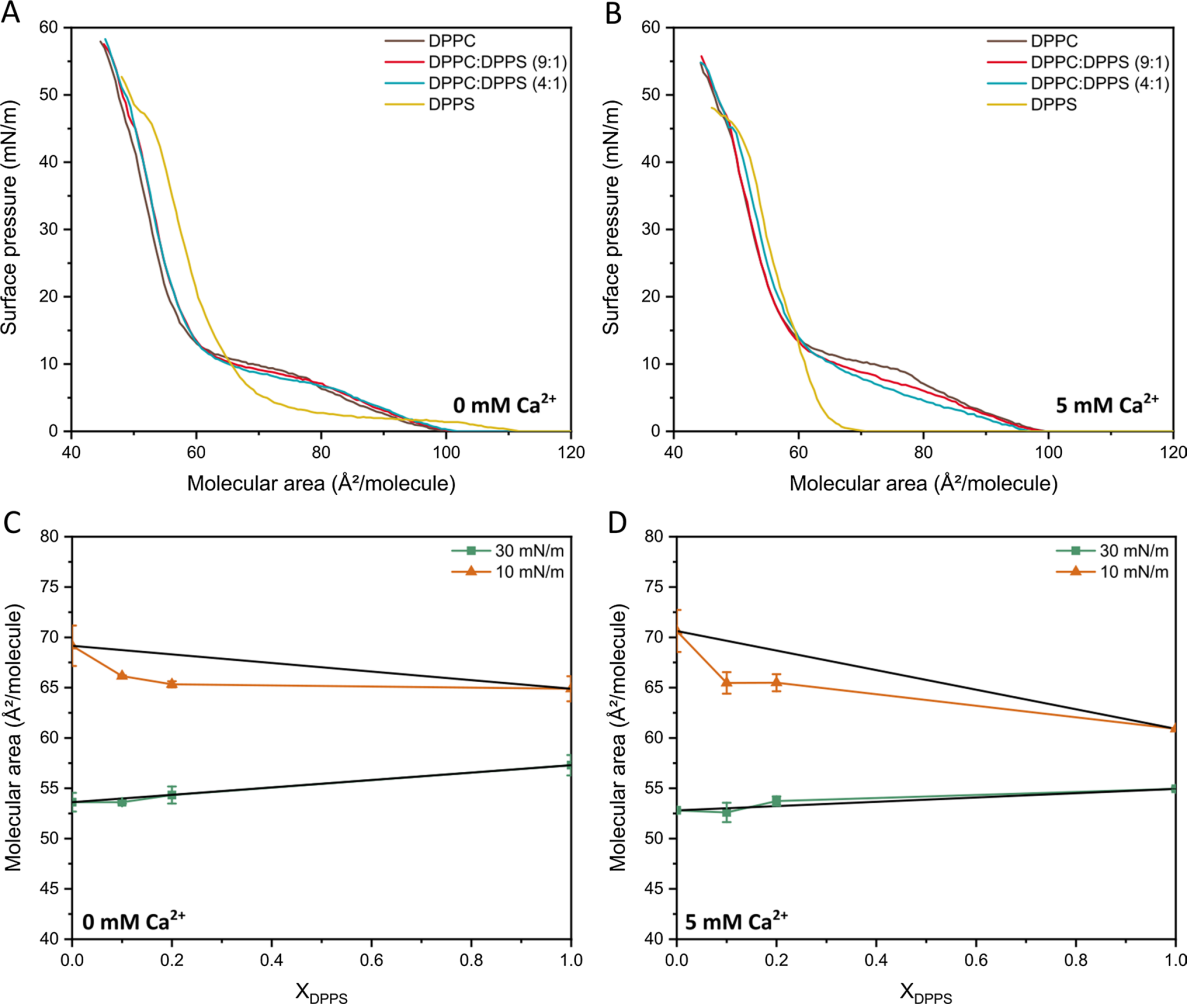
DPPC and DPPS organization at the air-liquid interface studied by means of Langmuir monolayers. Isotherms of Langmuir monolayers made of pure DPPC (brown lines), a mixture of DPPC and DPPS at a molar ratio of 9:1 (red lines) and 4:1 (blue lines), and pure DPPS (yellow lines) in HBS in the absence (**A**) and in the presence (**B**) of 5 mM of Ca^2+^. The mixture behavior was evaluated by deviations in the mean molecular area at the pressures of 10 and 30 mN/m from calculated ideal mixing (black lines in C and D), both in absence (**C**) and presence (**D**) of 5 mM Ca^2+^

The adsorption of annexin A5 on the lipid membranes and the formation of domains in the presence and absence of Ca^2+^ was investigated by means of Langmuir monolayers. Figure 3 shows the surface pressure (*π*) versus surface area (A) isotherms for Langmuir monolayers made of DPPC, DPPS and a mixture of DPPC and DPPS at 9:1 and 4:1 molar ratios, both in the presence and in the absence of Ca^2+^.

The isotherm of the monolayers made of pure DPPC (Fig. 3A, brown line) displayed the typical profile previously reported in the literature (Ross et al. 2001; Ruiz et al. 2017). The plateau corresponding to a liquid condensed/liquid expanded phase coexistence can be observed at *π* ~ 10 mN/m (McConlogue et al. 1998). The addition of DPPS to the monolayers did not cause any significant change in the profile of the isotherm (Fig. 3A, red and blue lines), which can be assigned to the fact that DPPC and DPPS have hydrocarbon chains of similar lengths. The isotherm of the monolayers made of pure DPPS also exhibited a typical profile, yet it had a lower minimum molecular area than the monolayers made of pure DPPC due to the smaller size of the polar head of DPPS (Ross et al. 2001). The addition of 5 mM Ca^2+^ to the subphase changed the lipid organization at the interface as demonstrated by the changes in the profile of the isotherms, especially below 10 mN/m, in which the liquid expanded to liquid condensed phase transition occurs (Fig. 3B). Concentrations Ca^2+^ lower than 5 mM Ca^2+^ did not significantly affect the behavior of the monolayers (see Supplementary Information). In the presence of Ca^2+^, the *π* value at the plateau assigned to the liquid condensed/liquid expanded phase coexistence increased for the monolayers made of pure DPPC (Fig. 3B, brown line), while it decreased for the monolayers made of a mixture of DPPC and DPPS (Fig. 3B, red and blue lines). Moreover, the plateau disappeared in the isotherm of the monolayers made of pure DPPS and the minimum molecular area was displaced towards lower values due to the shielding effect of Ca^2+^ (Fig. 3B, yellow line) (Ross et al. 2001).

Due to its negatively charged polar head, DPPS has greater affinity for Ca^2+^ than DPPC. The values of the molecular area for the monolayers made of a mixture of DPPC and DPPS showed a negative deviation at π = 10 mN/m (phase transition) compared to the ideal mixing represented by the solid black lines (Fig. 3C, D).

Liquid condensed domains dispersed in a liquid expanded phase is the main feature of the phase transition at π = 10 mN/m (Derradi et al. 2019). The reduced area indicates that the attraction between DPPC and DPPS molecules is predominant in the condensed domains. No deviation regarding the ideal behavior was observed at higher values of π (e.g., 30 mN/m), probably due to the highly compact organization at this stage. The addition of Ca^2+^ promoted a reduction in the π of the plateau assigned to the phase transition in DPPS-containing monolayers (Fig. 3B). Although this behavior might arise from the attraction between the lipid molecules favored by Ca^2+^, thus lowering the surface activity of condensed domains, we did not observe significant differences related to the thermodynamic parameters of mixing for these monolayers (Fig. 3D), indicating a more intricate organization of the membrane at this stage.

**Fig. 4:**
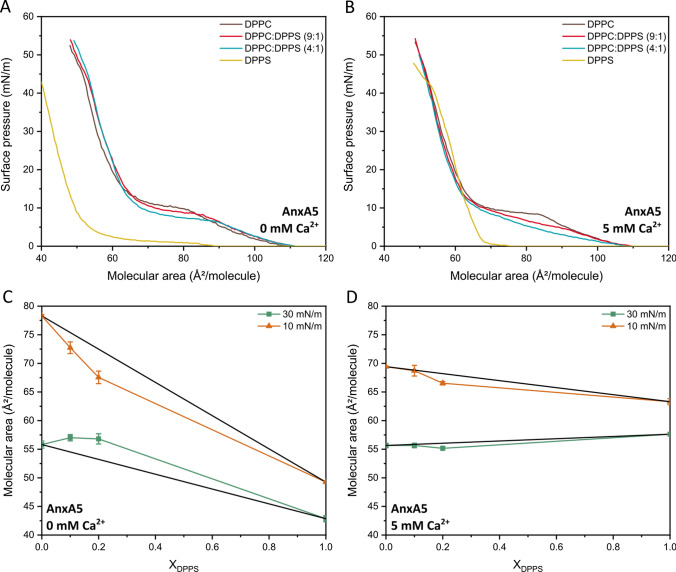
Interaction of DPPC and DPPS with AnxA5 in Langmuir monolayers either in the or in the absence of Ca^2+^. **A **and **B** Isotherms of Langmuir monolayers made of pure DPPC (brown lines), a mixture of DPPC and DPPS at a molar ratio of 9:1 (red lines) and 4:1 (blue lines), and pure DPPS (yellow lines) in HBS in the presence of annexin A5 in the absence or presence of 5 mM of Ca^2+^. **C **and **D** Mean molecular area was calculated from a ideal mixing (black line) to a mixture of DPPC and DPPS in the presence of annexin A5 in the absence and in the presence of 5 mM of Ca^2+^

The role of annexin A5 and Ca^2+^ in the lipid organization at the air-liquid interface was also investigated (Fig. 4). The addition of annexin A5 led to a shift toward higher values of the area of the isotherms for the monolayers made of pure DPPC and a mixture of DPPC and DPPS, irrespective of the presence of Ca^2+^. Conversely, for the monolayers made of pure DPPS the addition of annexin A5 led to a shift toward lower values of the area of the isotherms, suggesting that the protein shielded the charges of the lipids’ polar heads and promoted the compaction of the monolayers, even in absence of Ca^2+^. Having an isoelectric point of 4.93 (Köhler et al. 1997), annexin A5 is positively charged at the pH 7.4 of the subphase, which may explain the attraction to the negatively charged polar head of DPPS (Mukhopadhyay and Cho 1996). The way by which the annexin A5 interacts with anionic membranes was dependent on the presence of Ca^2+^ in the subphase (Fig. 4B).

The lower values of molecular area found at π = 10 mN/m compared to the theoretical values was indicative of the role of attractive interactions in the stabilization of condensed domains, especially in the absence of Ca^2+^. However, the increase in the molecular area at π = 30 mN/m in absence of Ca^2+^ suggested that the protein penetrated in the monolayers (Ma et al. 2020). This could also suggest that the domains able to either stabilize or destabilize the membrane in terms of positive or negative deviations from ideal mixture behavior might be enriched in DPPS molecules, which also promotes the adsorption of annexin A5 at specific sites of the membrane.

#### Stability of the monolayers in the presence of annexin A5

**Fig. 5:**
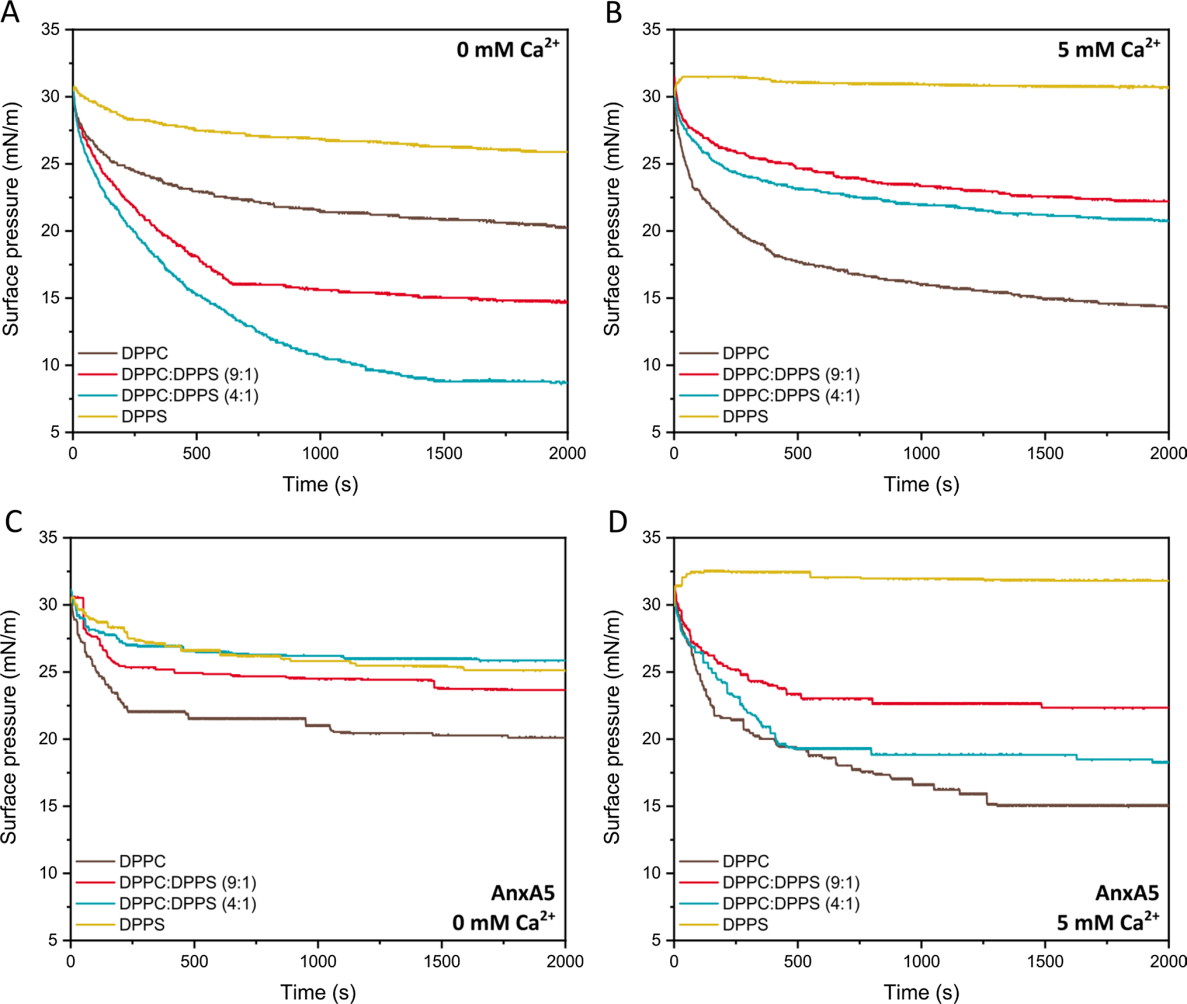
Role of Ca^2+^ and annexin A5 in the stability of lipid monolayers. Surface pressure changes for lipid monolayers made of pure DPPC (brown lines), a mixture of DPPC and DPPS at a molar ratio of 9:1 (red lines) and 4:1 (blue lines), and pure DPPS (yellow lines) in the absence (**A**, **B**) and in the presence (**C**, **D**) of annexin A5 and in the absence (**A**, **C**) and in the presence (**B**, **D**) of 5 mM of Ca^2+^. The starting pressure was fixed at 30 mN/m and recorded as a function of time until no significant variation was observed. Mean destabilization rates are shown in (**E**)

The changes in the value of π with time, starting at π = 30 mN/m, were recorded to better describe the role of annexin A5 and Ca^2+^ in the stability of lipid monolayers at surface pressures close to those found in cell membranes (Marsh 1996). The decrease in the value of π for the monolayers made of pure DPPS was the lowest among the monolayers tested (Fig. 5A, yellow line), which suggested a greater stability of the monolayers made of pure DPPS. The addition of Ca^2+^ further stabilized the monolayers made of pure DPPS (Fig. 5B, yellow line), due to the shielding effect of the ions bound to the negatively charged polar heads of the lipids. The monolayers made of a mixture of DPPC and DPPS also displayed a greater stability in the presence of Ca^2+^, which supports the relevance of Ca^2+^ binding (Fig. 5B).

Table 1 shows the rate of the π changes calculated from time (t) t = zero to t = 250 s [dπ/dt]. The stability of the monolayers was also assessed by the value of Δπ, calculated as the difference between the value of π at t = 0 s and t = 2000s. Higher values of dπ/dt and/or Δπ are indicative of monolayers with a lower stability.

**Table 1:** Role of calcium and annexin A5 in the stability of monolayers made of DPPC, DPPS and a mixture of DPPC and DPPS

Composition	Ca^2+^	AnxA5	dπ/dt (× 10^-2^)	Δπ
DPPC	–	–	– 1.79 ± 0.01	10.2 ± 0.03
	+	–	– 3.03 ± 0.02	16.1 ± 0.04
	–	+	– 2.98 ± 0.02	9.9 ± 0,08
	+	+	– 3.82 ± 0.01	15.1 ± 0.07
DPPC:DPPS (9:1)	–	–	– 3.78 ± 0.02	15.6 ± 0.09
	+	–	– 1.50 ± 0.01	7.8 ± 0.04
	–	+	– 2.45 ± 0.02	6.4 ± 0.05
	+	+	– 2.03 ± 0.01	7.6 ± 0.06
DPPC:DPPS (4:1)	–	–	– 3.54 ± 0.03	21.5 ± 0.11
	+	–	– 1.94 ± 0.01	10.8 ± 0.07
	–	+	– 1.19 ± 0.01	4.2 ± 0.02
	+	+	– 2.50 ± 0.01	11.7 ± 0.09
DPPS	–	–	– 0.95 ± 0.01	4.4 ± 0.03
	+	–	0	0
	–	+	– 1.01 ± 0.01	4.9 ± 0.03
	+	+	0	0

First derivative of π vs time calculated from time (t) t = zero to t = 250 s [dπ/dt] and variation of the value of π between t = 0 s and t = 2000s (Δπ) calculated from the isotherms showed in Fig. 5. *AnxA5* annexin A5, *DPPC *1,2-dipalmitoyl-*sn*-glycero-3-phosphatidylcholine, *DPPS* 1,2-dipalmitoyl-*sn*-glycero-2-phosphatidylserine.

The addition of Ca^2+^, Annexin A5, and both to the monolayers made of pure DPPC led to a decrease in the monolayer stability, however it led to an increase in the monolayer stability when added to the monolayers made of pure DPPS or a mixture of DPPC and DPPS. It is worth noting that the addition of Ca^2+^ stabilized the monolayers made of DPPC and DPPS with a molar ratio of 9:1 more than the addition of annexin A5. However, for the monolayers containing a higher amount of DPPS (i.e., with a DPPC:DPPS molar ratio of 4:1, Fig. 5C) the stabilizing effect of annexin A5 was greater than that of Ca^2+^ alone and both Ca^2+^ and annexin A5. These data highlighted the stabilizing effect of annexin A5 on the lipid monolayers in the presence of DPPS.

### Fluorescence microscopy

**Fig. 6:**
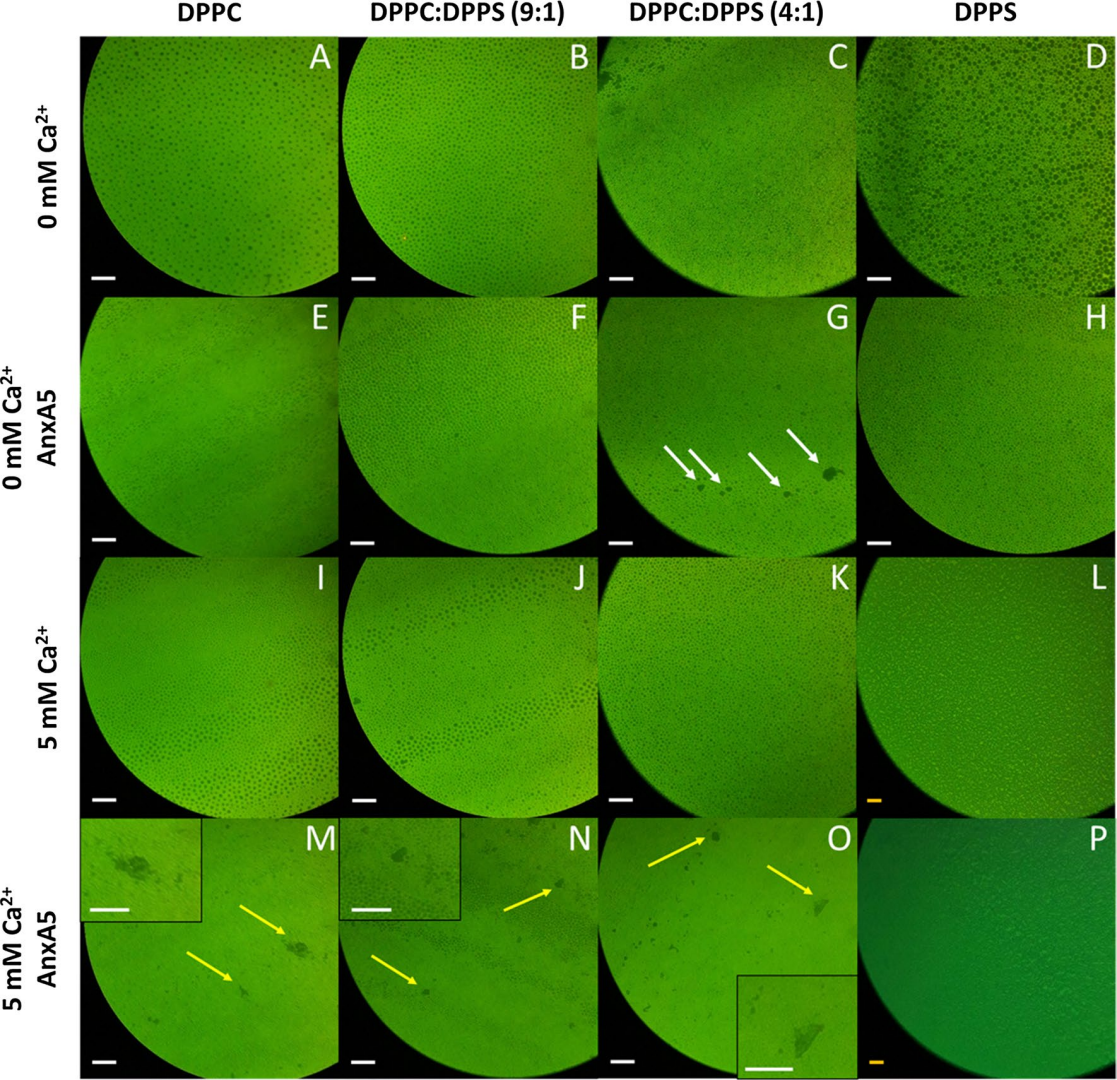
Formation of domains in lipid monolayers. Fluorescence microscopy of lipid monolayers made of pure DPPC (**A**, **E**, **I**, **M**), a mixture of DPPC and DPPS at a molar ratio of 9:1 (**B**, **F**, **J**, **N**) and 4:1 (**C**, **G**, **K**, **O**), and pure DPPS (**D**, **H**, **L**, **P**) at a surface pressure of 10 mN/m in the absence (**A**–**H**) and in the presence (**I**–**P**) of 5 mM of Ca^2+^ and in the absence (**A**–**D** and **I**–**L**) and in the presence (**E**–**H** and **M**–**P**) of annexin A5 (100:1 lipid:protein molar ratio). Yellow arrows point to larger condensed domains assigned to the presence of the protein. White scale bar: 40 µm; Orange scale bar: 80 µm

Figure 6 shows the fluorescence images of the monolayers at the liquid condensed/liquid expanded coexistence phase (π = 10 mN/m). The goal of assessing the membrane at this stage was to see if annexin A5 could also induce lipid phase transition rather than just bind to it at the condensed phase (30 mN/m). The darker areas in the images correspond to the condensed domains due to the insolubility of the dye NBD-HPC is such environment (Okonogi and McConnell 2004; Derradi et al. 2019). The presence of liquid condensed circular domains imaged for the monolayers made of pure DPPC and pure DPPS are consistent with previously reported data (McConlogue and Vanderlick 1997; Ross et al. 2001; Shieh and Zasadzinski 2015). The addition of DPPS did not affect the size nor the distribution of the domains (Fig. 6B).

The addition of annexin A5 affected the distribution of these domains by enabling the formation of larger domains with an irregular shape, probably due to the protein adsorption (Fig. 6E-H), in special at higher DPPS concentrations (write arrow, Fig. 6G). We have found that the addition of Ca^2+^ promoted slight changes in the size and distribution of the condensed domains, which corroborate the reduction of π observed at the liquid expanded/liquid condensed plateau in the Langmuir monolayers composed by DPPS (Fig. 3B). The addition of Ca^2+^ also enhanced the distribution of irregular domains promoted by annexin A5, both in terms of domains shape and size (Fig. 6M–O). This effect was also observed for the monolayers made of pure DPPC and a mixture of DPPC and DPPS (Fig. 4), making it difficult to correlate the observed changes to specific interactions between annexin A5 and DPPS molecules, even though our differential scanning calorimetry and Langmuir monolayer data would suggest such association. Interestingly, the fluorescence images obtained for the monolayers made of pure DPPS in the presence of annexin A5 and Ca^2+^ at the subphase were like those obtained only in the presence of Ca^2+^, suggesting that the addition of annexin A5 was not able to restore the disruption of the domains by Ca^2+^ in these monolayers (Fig. 6P).

## Conclusions

Langmuir monolayers were used as a biomimetic membrane model to show the effect of Ca^2+^ and annexin A5 in the organization of lipid membranes containing DPPS. The ion stabilizes lipid domains enriched in DPPS by reducing charge repulsion. The adsorption of annexin A5 on the monolayers seemed to increase the heterogeneity of the lipid domains, supporting the model of formation of DPPS-enriched domains (Bouter et al. 2011, 2015). At values of surface pressure of biological relevance (i.e., 30 mN/m), we observed that the formation of lipid domains destabilized DPPS-containing monolayers, leading to their fusion in the absence of annexin A5, supporting the stabilizing effect of the protein. These data shed light on the role of annexin A5 in the repair of membrane defects (Bouter et al. 2011). Mechanistically, the local increase in the concentration of Ca^2+^ at the defect sites of damaged cell membranes (Steinhardt et al. 1994) would induce the formation of domains enriched in negatively charged lipids, to which annexin A5 could adsorb, forming bidimensional arrays and avoiding the further expansion of the defect (Oling et al. 2001). We can speculate that the role of annexin A5 in matrix vesicles might be to stabilize the vesicles’ membrane during the accumulation of Ca^2+^ and phosphate in their lumen, thus avoiding the premature disruption of their membrane (Cruz et al. 2020). Our study could help to explain why matrix vesicles carry an unexpectedly high amount of annexin A5, a topic that has intrigued researchers on this field for decades (Wuthier and Lipscomb 2011).

### Supplementary Information

Below is the link to the electronic supplementary material.

Supplementary file1 (DOCX 274 KB)

## Data Availability

The data is in our possession and if requested, with justification, it can be provided.
