# Supplementary material for: Annexin A5 stabilizes matrix vesicle-biomimetic lipid membranes: unravelling a new role of annexins in calcification
Source: Eur Biophys J. 2023 Nov 8;52(8):721–33. doi: 10.1007/s00249-023-01687-4 (PMC10682239; doi:10.1007/s00249-023-01687-4)
Supplement: Supplementary file 1 — Supplementary file1 (DOCX 274 KB) [file 249_2023_1687_MOESM1_ESM.docx]

**SUPLEMENTAR INFORMATION**


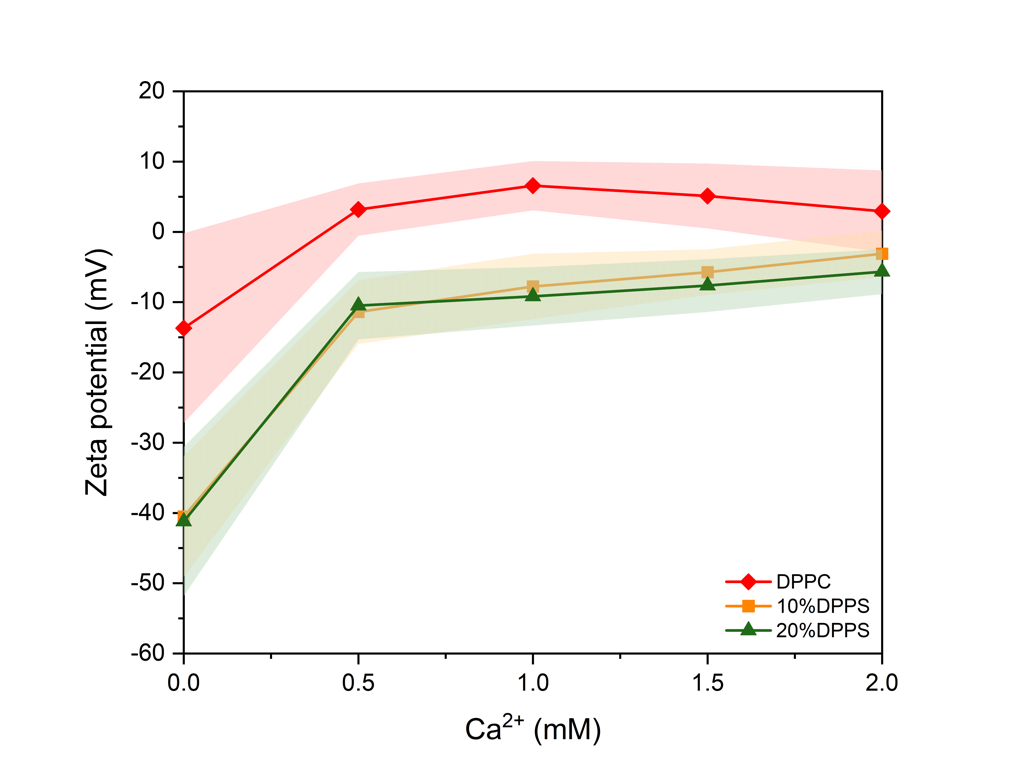


**Figure S01.** Zeta potential distribution of liposomes constituted by DPPC, DPPC:DPPS (9:1) [10%], and DPPC:DPPS(4:1) [20%] during aggregation assay.


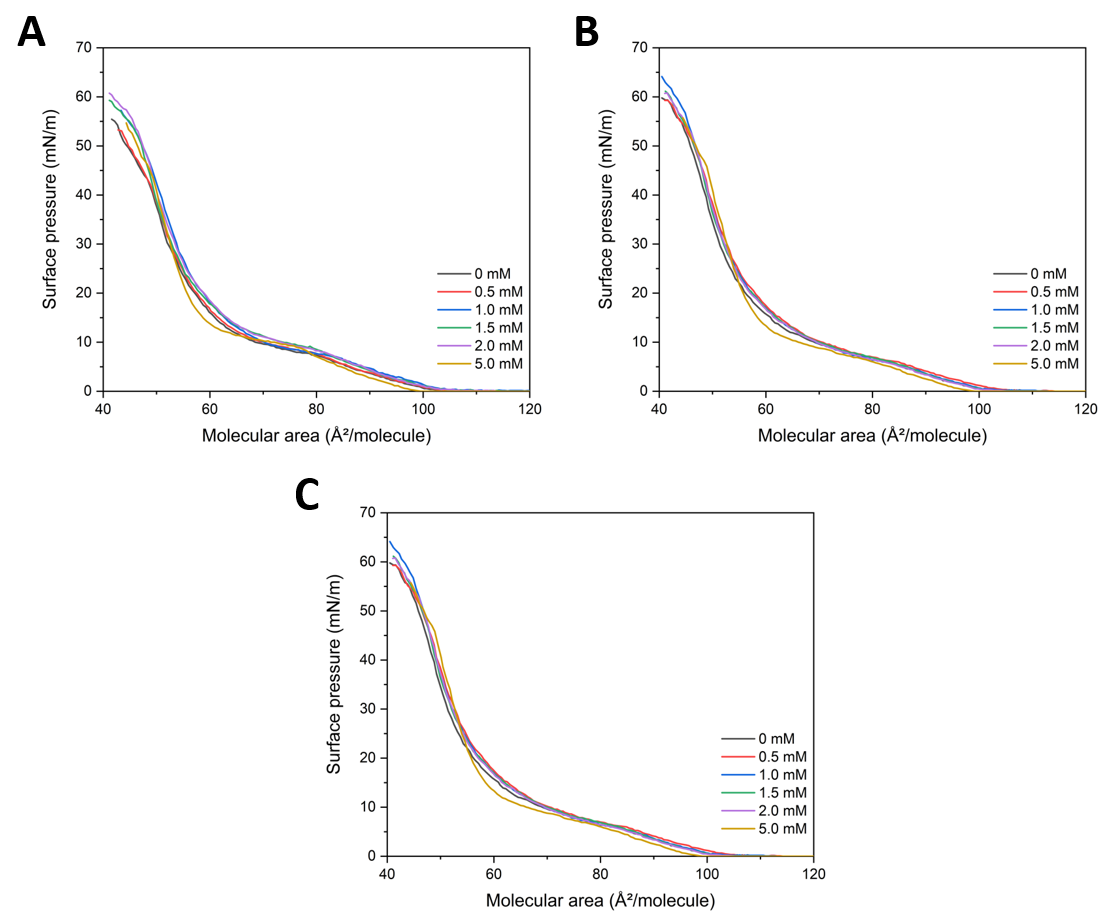


**Figure S02.** Effect of calcium concentration over the monolayers constituted of pure DPPC (A); DPPC:DPPS (9:1) (B); and DPPC:DPPS (4:1) (C).
